# Pathological Myopia Image Recognition Strategy Based on Data Augmentation and Model Fusion

**DOI:** 10.1155/2021/5549779

**Published:** 2021-05-05

**Authors:** Jianfeng Cui, Xiaoyun Zhang, Feibing Xiong, Chin-Ling Chen

**Affiliations:** ^1^School of Software Engineering, Xiamen University of Technology, Xiamen 361024, China; ^2^School of Opto-electronic and Communications Engineering, Xiamen University of Technology, Xiamen 361024, China; ^3^School of Computer and Information Engineering, Xiamen University of Technology, Xiamen 361024, China; ^4^School of Information Engineering, Changchun Sci-Tech University, Changchun 130600, China; ^5^Department of Computer Science and Information Engineering, Chaoyang University of Technology, Taichung 41349, Taiwan

## Abstract

The automatic diagnosis of various retinal diseases based on fundus images is important in supporting clinical decision-making. Convolutional neural networks (CNNs) have achieved remarkable results in such tasks. However, their high expression ability possibly leads to overfitting. Therefore, data augmentation (DA) techniques have been proposed to prevent overfitting while enriching datasets. Recent CNN architectures with more parameters render traditional DA techniques insufficient. In this study, we proposed a new DA strategy based on multimodal fusion (DAMF) which could integrate the standard DA method, data disrupting method, data mixing method, and autoadjustment method to enhance the image data in the training dataset to create new training images. In addition, we fused the results of the classifier by voting on the basis of DAMF, which further improved the generalization ability of the model. The experimental results showed that the optimal DA mode could be matched to the image dataset through our DA strategy. We evaluated DAMF on the iChallenge-PM dataset. At last, we compared training results between 12 DAMF processed datasets and the original training dataset. Compared with the original dataset, the optimal DAMF achieved an accuracy increase of 2.85% on iChallenge-PM.

## 1. Introduction

Pathologic myopia (PM) is one of the major causes of visual impairment worldwide [1–3]. As myopia deepens, it is often accompanied by deforming changes in the posterior pole of the eye [3]. These changes are usually manifested as sclerotic atrophy, choroidal atrophy, and growth of the ocular axis, which may be associated with various complications of the eye, such as amblyopia, glaucoma, cataracts, vitreous clouding, and retinal detachment [1]. Complications of pathological myopia are considered to be the main reasons for visual impairment and blindness today, particularly in East Asia [4, 5]. Pathological myopia causes visual impairment due to various pathologies of the macula, peripheral retina, and optic nerve. Structural deformities of the eye, including posterior sclerite uveitis, may accelerate the progression of these diseases [1, 3, 6–9].

According to a summary of 145 studies regarding the global prevalence of myopia and PM, there are approximately 1950 million people with myopia (accounting for 28.3% of the global population) and 277 million people with PM (accounting for 4.0% of the global population), and these numbers are predicted to increase to 4758 million (accounting for 49.8% of the global population) for myopia and 938 million (accounting for 9.8% of the global population) for PM by 2050 [5].

The prevalence of high myopia and pathological myopia shows an increasing yearly trend due to the changes in environmental factors and lifestyle [10–14]. In China, the number of ophthalmologists differs significantly from that in developed countries, but the demand for ophthalmologists in China is already huge; with the growth of social aging, the number of ophthalmologists has been rising in recent years [10, 12, 14]. In consequence, the visual impairment caused by pathological myopia complications will become increasingly serious in the coming decades. The people's growing medical needs contrast sharply with the increasingly deficient medical resources in the current medical field.

On the one hand, the problem of “difficult and expensive access to medical care” still exists, which is mainly caused by the severe scarcity of talented physicians and the long training period for doctors. On the other hand, with the health problems gradually worsening, people are paying more and more attention to their health, which aggravates the demand for medical services. This is a social problem to which AI technology can offer the medical industry a solution [15–18].

Over the past two decades, with the development of imaging techniques, such as optical coherence tomography, frequency-domain OCT, and 3D magnetic resonance imaging, the complications associated with high myopia have been well known [16, 19–23]. For example, the optic nerve, macula, and neoplastic lesions can be magnified by OCT to extraordinary resolution for evaluation. In addition, myopic retractile macular lesions and domed macular lesions can be utilized in the same way. The advent of new therapies, including antineovascular drug therapies and vasectomy, has led to an improved prognosis for some of the complications associated with high myopia.

Medical artificial intelligence (AI) technologies have been well developed in recent years [20]. A case in point is the application of machine learning-based AI technology to ophthalmology [20, 21]. The diagnosis of many eye diseases relies heavily on the results of ophthalmic examinations, most of which are built on imaging studies. Eye images are delicate, complex, and informative, and diagnostic results are heavily dependent on the doctor's knowledge and clinical experience, which makes diagnosis subjective and time-consuming [24, 25]. The development of medical AI has significantly improved the efficiency of ophthalmic disease diagnosis in clinical work and reduced the burden on ophthalmologists [20–22].

CNNs, which are data-driven and can automatically extract relevant features, have secured better results in image recognition than traditional methods [26]. Therefore, it is considered to be a new choice to introduce CNN techniques into medical image processing. Lately, many studies have been conducted on this matter and applied CNNs to fundus image recognition, whose results generally surpass those based on traditional recognition ways [19, 26–31].

Although the CNN-based fundus image recognition method outperforms traditional methods to a certain extent, there are still some problems. For example, the amount of medical image data is large, while the number of positive samples is small. Nevertheless, a common fact is that the training process of the model mainly depends on the dataset, namely, the training effect of the model can be well improved by the dataset after DA processing [32].

Furthermore, with the continuous development of deep learning in the field of images, CNNs have been increasingly complicated. Each model has its unique advantages, but it is not guaranteed that every aspect of the model will perform well. For instance, the model's lack of expressive ability will lead to the weakness in the recognition of some rare lesion images [32]. To address this problem, researchers have proposed optimized neural network models from different perspectives and achieved effective results. However, there are a lot of difficulties in enhancing the existing models. For example, when the researchers optimize the models (such as widening and deepening models), they cannot predict the effectiveness of the models but just observe whether the optimization operation improves the performance of the original models through the training results. Besides, even if such optimization is effective, it may be computational and time-intensive or needs a long development cycle, so it cannot address the problem effectively [33–35].

In order to deal with these issues, the main contributions of this paper are described as follows:

1. 12 DAMFs will be designed based on the iChallenge-PM dataset. To our knowledge, these DA methods cover all the operations used in the current DA. The purpose is to increase data characteristics, suppress sample imbalance, and effectively improve the quality of datasets.
2. Based on AlexNet, VGG-16, GoogLeNet, and ResNet-50 models, different optimizers, loss functions, and learning rates are constructed. Then, the model with the highest accuracy will be adopted as the primary learner and trained based on 12 datasets. The generalization ability of the model will be enhanced through this method.
3. The abovementioned primary learner prediction will be used as a new input and put into the secondary learner, namely, the hard voting model, and then the fusion model is trained to form the final model.
4. The model optimized by the above operations achieves high accuracy without transfer learning. More importantly, by using the augmented dataset and the model fusion method, we effectively avoid overfitting and improve the generalization ability of the model when processing various data, which further improves the expressive ability of the model. As a result, the accuracy of the model in recognizing complex and rare case images will be effectively improved.

## 2. Literature Review

In this section, we mainly reviewed the related literature on automatic disease diagnosis from fundus photography and DA.

### 2.1. Automatic Disease Diagnosis from Fundus Photography

Most of the conventional ophthalmic diseases can be examined from fundus photography, including PM, age-related macular degeneration (AMD), diabetic retinopathy (DR), and glaucoma. Conventional diagnosis methods tremendously depend on doctors' professional experience and knowledge, which results in a high misdiagnosis rate and a huge waste of medical data [36, 37]. The deep integration of ophthalmology and AI offers potential for revolutionizing current disease diagnosis patterns and generating a significant clinical impact. As for PM classification, Freire et al. employed Xception as the baseline architecture with ImageNet pretrain weights to diagnose PM from fundus images [38]. Zhang et al. used the feature selection of PM valuable information in the images to improve the training effect of the model [31]. They demonstrated that the new method was much efficient by using less than 25% of the initial candidate feature set. Not only in the field of image classification but DA also occupies a highly important position in the field of target detection. Sun et al. put forward two new DA modules consisting of channel-wise random Gamma correction and channel-wise random vessel augmentation [27]. They argued that their method could ameliorate the performance and robustness of a classic CNN architecture. However, most medical imaging samples are unbalanced. Although there may be a large number of samples, the types of samples are limited. Different from previous works, in this paper, we focused on the first stage of deep learning, which was DA. And by means of this strategy, the defect of data imbalance could be effectively solved and the overfitting in the training process could be suppressed.

### 2.2. Data Augmentation

DA highlights the characteristics of image data and prevents overfitting in the training effect [33]. In existing studies, researchers divide DA methods into the standard method, data disrupting method, data mixing method, DA method based on reinforcement learning, and fusion-based image augmentation method.

1. *Standard Data Augmentation Method.* AlexNet [39] preprocesses the data using random cropping and horizontal flipping, and it has been verified on CIFAR-10. Random cropping prevents CNN from overfitting specific features by changing the obvious features in the image. Facebook artificial intelligence research uses another color translation method, called color dithering, to improve the process of ResNet [40]. Color dithering randomly changes the brightness, contrast, and saturation of the image. These DA techniques play an important role in the training of a model. Fu et al. performed extensive experimentation on a large set of images with varying illuminations [41]. The performance is analyzed both quantitatively and qualitatively. However, as the number of parameters increases, the risk of overfitting also increases. Many studies have proposed more complex CNNs. Therefore, more powerful DA strategies are particularly important.
2. *Data Disrupting Method*. [42] Contrary to (1), a data disrupting method produces unnatural images by destroying images' features. Dropout on the input layer is a DA technique that disturbs and masks the original information of given data by dropping pixels. Pixel dropping functions as injection of noise into an image. It makes the CNN robust to noisy images and contributes to generalization rather than enriching the dataset, randomly erasing a region in an image at every training step. It is an extension of dropout, where the masking of regions behaves like injected noise and makes CNNs robust to noisy images. Under this condition, CNNs need to learn other parts that are usually ignored.
3. *Data Mixing Method*. This is a special case of (2), where a mixup alpha blends two images to construct a new training image [43]. Mixup can train CNNs on convex combinations of pairs of training samples and their labels and enables CNNs to favor a simple linear behavior in-between training samples. This behavior makes the prediction confidence transit linearly from one class to another class, thus providing smoother estimation and margin maximization. Therefore, the mixup makes CNNs robust to adversarial examples and stabilizes the training of generative adversarial networks.
4. *Data Augmentation Method Based on Reinforcement Learning* [44]. Autoenhancement is a framework based on reinforcement learning to explore the optimal augmentation combination [45]. Hence, it is not a DA method but is an external framework. It achieves significant results during the CIFAR-10 classification and proves the contribution of reinforcement learning to DA research.
5. *Fusion-Based Image Augmentation Method*. Fusion is preferred to the direct application of traditional techniques since it involves an amalgamation of traditional techniques, rather than the application of a single technique. Fusion can be done in various ways, out of which multiscale fusion has proved to be one of the best. Parihar et al. presented a detailed analysis of image enhancement techniques based on multifusion, thereby giving an insight into the algorithm used in each method, along with its implementation framework [36, 42].

In this paper, we adopted the concept of (5) and integrated the image enhancement techniques of (1), (2), and (3), aiming to enlarge the features of images from the perspective of color, direction, shape, and so on.

## 3. Materials and Methods

In this session, we first introduced the dataset of iChallenge-PM [46] composed of 1200 annotated retinal images. In another part of this section, we introduced the primary learner, which included some classical convolutional neural networks and corresponding components. These frameworks have become general arrangements for image classification and have been extensively utilized in different computer vision tasks.

### 3.1. iChallenge-PM

The iChallenge-PM dataset contained 1200 annotated color fundus photos with non-PM (50%) and PM (50%) cases. Specifically speaking, the dataset contained 400 images in the training set, validation set, and test set, respectively, while the test set was not public. We reclassified the 800 public image data and divided the validation set into a new validation set and a test set according to the ratio of 50% : 50%. All images data were reshaped to a size of 224 ^∗^ 224 before using DA. Under the partitioning of iChallenge-PM, the reference standard of PM presence was obtained from the health records, which was not based solely on fundus image but also took OCT, visual test, and other factors into consideration. For the training data, PM, HM, and normal labels were reflected in the image file names, with 0 denoting normal cases while 1 denoting abnormal cases. However, we did not utilize any human-annotated labels during network training. To evaluate the effectiveness of our method, we employed accuracy and loss rate as the evaluation indices. Accuracy was often the most important index for doctors and patients.

### 3.2. Classifiers and Components

**Figure 1:**
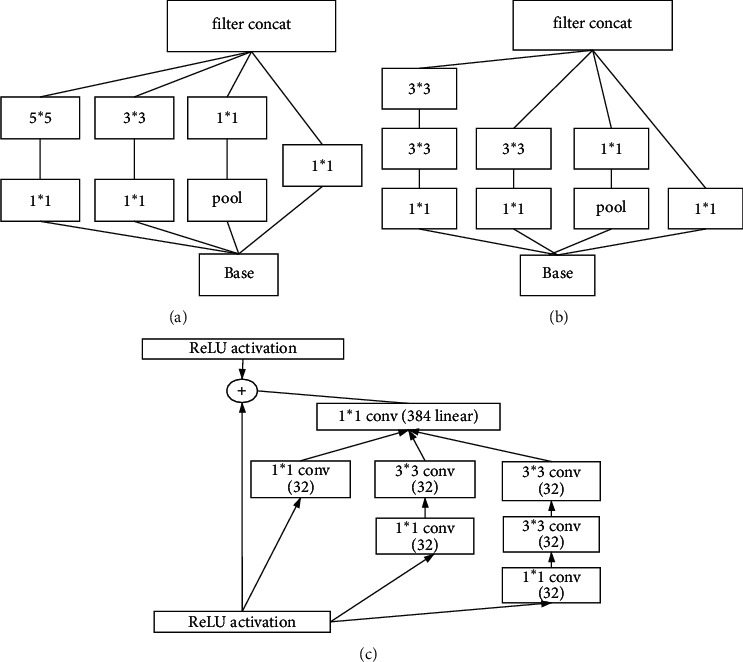
Evolution of inception structures: (a) Inception-V1 chart; (b) Inception-V2 chart; (c) Inception-V3 chart.

**Figure 2:**
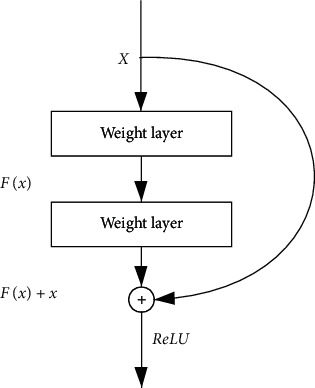
Residual structures in the ResNet network.

**Figure 3:**
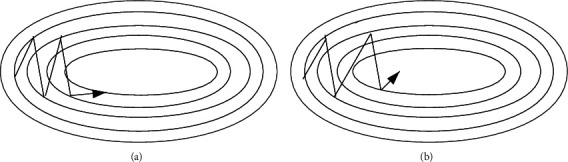
Source: Genevieve B.Orr: (a) SGD without momentum; (b) SGD with momentum.

(1) *AlexNet*. The AlexNet network proposed by Krizhevsky et al. was the first to use five convolutional layers and three fully connected expenses to achieve the classification of 1000 classes of images, thus becoming the seminal breakthrough in image classification based on deep learning. Compared with other traditional convolutional neural networks, AlexNet applied various methods to improve deep convolutional networks. For example, the rectified linear unit (ReLU) nonlinear activation function was used to speed up the training of the network, multi-GPU convolutional operations were implemented to address the limitations of insufficient graphic card resources at the time, and the DropOut random inactivation strategy was introduced to reduce overfitting at the full connection layer. Furthermore, strategies such as local response normalization, overlap pooling, as well as augmentation, were proposed by AlexNet to improve the classification and generalization capabilities of the model.
(2) *VGG*. The VGG network was proposed by Simonyan et al., in which filters of 5 ^∗^ 5 and 7 ^∗^ 7 were replaced by filters of 3 ^∗^ 3. It was a basic idea embodied by VGG that the receptive fields of multiple small convolutional layers in series could be in the same size as that of a large convolutional layer. For example, two 3 ^∗^ 3 convolutional fields in sequences had the same field size as one 5 ^∗^ 5 convolutional field, and their convolutional effects were the very same. However, multiple small convolutional layers concatenated together had fewer parameters and more nonlinear transformations. This was most effective in better learning the features. At the same time, VGG also increased the network structure to 16 or 19 layers. As the number of layers increased, the network enjoyed better feature representation and better model classification. Being simple and effective, VGG was still commonly used in the field of computer vision for image classification, detection, segmentation, super-resolution, and image styling.
(3) *GoogLeNet*. Google made a significant contribution to the development of deep convolutional neural networks through the proposed inception family. The most significant contribution of Inception-V1 (GoogLeNet) was to propose the inception structure while deepening the depth of the convolutional neural network. The structure increased the width of the network by concatenating multiple convolutional blocks of different sizes, which allowed the convolutional blocks to acquire information from discrete receptive fields. In addition, the structure took full advantage of the 1 ^∗^ 1 convolutional code to reduce a large number of network parameters, thereby improving the efficiency of computing resources. Inception-V2 proposed an excellent regularization method, namely, batch normalization, which made the data undergo a back normalization process before each convolution and was now the standard for deep convolutional networks. This approach was an excellent solution to the training problem of multilayered networks. The evolution of the inception structures is shown in Figure 1.
(4) *ResNet*. Kaiming He et al. established a deep residual network, namely, ResNet [14], which increased the network depth to 152 layers while ensuring the network accuracy and then further increased the depth to 1000 layers. Theoretically, the deeper the network was, the higher the accuracy should be. However, the authors experimentally found that blindly increasing the depth would lead to the degradation of the network when the depth reached a certain level. The gradient explosion and gradient disappearance of deep networks failed to train the model correctly and led to poor network performance. Inspired by highway network, the authors proposed the residual structure by adding a jump connection between the input and output of the convolutional block, which enabled the input to be passed directly to the output. The residual structure was essentially designed to learn a constant mapping with the nonlinear layer portion of the stack learning another mapping. As shown in Figure 2, if the residual structure was zero, we could easily train a constant mapping. In short, if a network could achieve the desired result by simply setting parameters manually, it was not difficult to train the network to converge to that result so that the added residual structure would at least not degrade the overall performance of the network. ResNet's residual module reduced the difficulty in training deep networks, solved the degradation problem well, and maximized the depth potential of convolutional networks. Eventually, ResNet outperformed human performance in terms of the ImageNet classification task for the first time.
(5) *Optimizer*. SGD [47] experienced difficulty exploring gorges. For example, territories, where the surface bent considerably more steeply in one measurement than in another, were basically around neighborhood optima. In these situations, SGD swayed over the gorge's slants and slowly advanced along the valley floor towards the local optimal direction, as indicated in Figure 3(a). The energy was a technique that quickened SGD and hose motions in a pertinent way, as shown in Figure 3(b). As suggested by formula (1), it achieved this by including a part *γ* of the update vector of the past time venture to the current update vector.
  (1) $$\begin{array}{c} v_{t}=\gamma v_{t-1}+\eta \nabla_{\theta }J\left(\theta \right),\;\text{where }\theta =\theta -v_{t}. \end{array}$$
The energy term *γ* was usually set to 0.9 or a comparative value. In general, when utilizing energy, we pushed a ball down a slope. The ball collected energy as it moved downhill, getting quicker and quicker in transit (until it arrived at its max speed, if there was air obstruction, for example, *γ* < 1). Something very similar happened to our boundary refreshing: the momentum term increases for the latitude where the gradient points in the same direction, while for the dimension where the gradient changes direction, the momentum term will decrease and update. Accordingly, we increased quicker union and diminished swaying.
(6) *Optimizer*. The logistic loss could be calculated by the following formula:
  (2) $$\begin{array}{c} \text{loss}=-\text{Labels}{}^{*}\;\log\left(\text{sigma}\left(X\right)\right)-\left(1-\text{Labels}\right){}^{*}\;\log\left(1-\text{sigma}\left(X\right)\right),\;\text{where sigma}\left(x\right)=\frac{1}{1+\exp\left(-x\right)}. \end{array}$$
After applying it to the above calculation, we got logistic loss formula as follows:
  (3) $$\begin{array}{c} \text{loss}=X-X{}^{*}\text{Labels}+\log\left(1+\exp\left(-X\right)\right). \end{array}$$
In order to calculate stability and prevent overflowing, the loss function would be calculated using the following formula:
  (4) $$\begin{array}{c} \text{loss}=\max\left(X,0\right)-X{}^{*}\text{Labels}+\log\left(1+\exp\left(-\;\left|X\right|\right)\right). \end{array}$$

### 3.3. Voter Model

Voting was a combination strategy aimed at classification problems in ensemble learning. The basic idea was to select the class with the highest output among all machine learning algorithms. There were two types of output judging from a machine learning classification algorithm: one was the direct output of class labels and another was the output of class probabilities. Using the former for voting was called majority/hard voting while using the latter for classification was called soft voting. Hard voting selected the label with the most output of the algorithm. If the number of labels was equal, the selection was made in ascending order. Soft voting used the class probabilities output by each algorithm to select a class. If the weight was input, a weighted average of the class probabilities of each class would be obtained, and the class with a large value would be selected. In this paper, our experiment used the hard voting mechanism.

### 3.4. Data Augmentation Strategy

**Figure 4:**
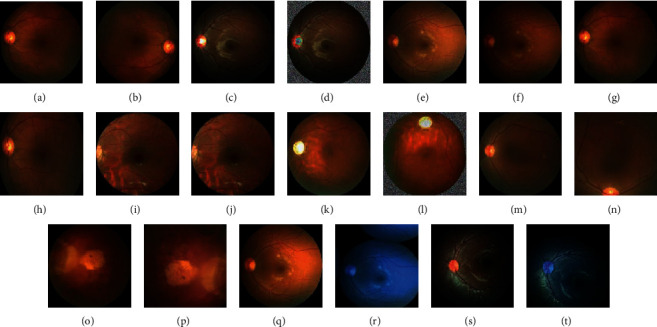
DA effect (original dataset image_x_: different images in the original dataset: (r), (s), and (t) were mutated from (q)). (a) Original dataset image_1_. (b) Randomly change direction. (c) Original dataset image_2_. (d) Randomly add Gaussian noise. (e) Original dataset image_3_. (f) random color. (g) Original dataset image_4_. (h) Random stretching. (i) Original dataset image_5_. (j) Randomly adjust the sharpness. (k) Original dataset image_6_. (l) Randomly flip, adjust colors, and add Gaussian noise. (m) Original dataset image_7_. (n) Randomly flip, adjust colors, and add Gaussian noise. (o) Original dataset image_8_. (p) Random stretching and cropping. (q) Original dataset image_9_. (r) Random cropping with 0–50 pixels around, 50% probability horizontal flip, and Gaussian blur (sigma = 0 to 3.0). (s) Original dataset image. (t) Randomly stack all operations.

Binary coding was used to represent positive and negative samples in this paper. Existing common DA methods consisted of randomly flipping the image (horizontally or vertically), randomly adding noise, rotating the image, changing the brightness, contrast, and saturation of the image, randomly cropping the image, randomly scaling/stretching the image, and randomly changing the clarity of the image. All these methods belonged to (1), (2), and (3) of Section 2.2. Based on these DA methods, we performed 12 different DAMF combinations and made 12 new datasets, as listed in Table 1. Specifically, third-party DA libraries were used in the 11th DAMF and 12th DAMF, respectively. As shown in Figure 4, all the images in the original dataset represented images without corresponding augmentation. The enhanced images were displayed following each original image. Figure 4(b) shows the randomly rotated operation, with the rotation angle at 90/180/270/360 degrees. Figure 4(d) shows the consequence of randomly adding Gaussian white noise to the original operation. Figure 4(f) describes the operation after random adjustment of brightness, saturation, and contrast built on the original image. Figure 4(h) describes the random cropping and stretching based on the original image. Figure 4(j) displays the image after randomly adjusting the sharpness. Figure 4(l) displays the image after randomly adjusting the contrast, saturation, and brightness on the original image and adding random Gaussian white noise. Figure 4(n) shows a randomly rotated, cropped, and stretched image built on the original image. Figure 4(p) shows the effect of randomly superimposing the images after all operations on the original images. Figure 4(r) shows the effect of utilizing the third-party library imaging to mutate the image. Figure 4(t) shows the effect of randomly superimposing all the above special effects.

### 3.5. Primary Learner Model

AlexNet, GooLeNet, VGG-16, and ResNet-50 were used as the primary learners in this paper. The experiment set the learning rate to 0.001 and utilized the optimizer and loss function described above. Each model was formed for 30 epochs, with each epoch covering all images in the training set.

The highest accuracy of each model was selected after comprehensive training, and the corresponding model parameters were saved. Then, the convolution layer of all primary learners would be frozen, which meant that the data could only be transmitted forward instead of backward after entering the primary learners.

### 3.6. Staking Model Integration Strategy and Hard Voting Model

Staking, as a hierarchical model integration framework, was one of the main strategies widely used in model integration. Taking two layers as an example, the first layer consisted of multiple base learners, and the original training set was the input of the primary learners. The output of the primary learners was treated as the secondary learners' input, which was the training set of secondary learners. The secondary learners continued training on the above training set to obtain the complete staking model.

As shown in Algorithm 1, processes 1–3 constructed the trained primary learners. Processes 5–9 were the prediction results of the training set using the trained primary learners, and this prediction was used as the training set for the secondary learners. Process 11 used the prediction results of the primary learners to train the secondary learners to get the fusion model.

**Figure 5:**
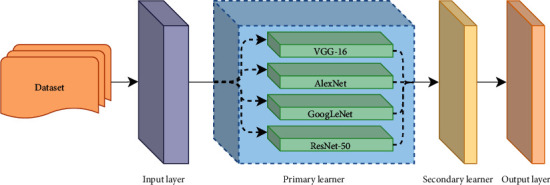
Logic diagram of the fusion model.

The design facilitated the extension of the model. In other words, the hard voting model could be replaced with other secondary learners based on different datasets.  Figure 5 displays the framework of the fusion model in this paper. First, the training set normalized the data through the input layer, namely, processed it into a format of the same size (224 ∗ 224). Next, the images were input into each classifier, respectively, in the primary learner. The classifier performed 30 times epoch supervised training on the image according to the label of all inputs and then used the training result as the input of the secondary learning, and the final classification result was obtained after voting.

## 4. Results

### 4.1. Lab Environment

Hardware environment is as follows: CPU 4 cores, RAM 32 GB, GPU v100, video memory 16 GB, and disk 100 GB.

Environment configuration is as follows: Python version python3.7 and framework version PaddlePaddle 1.8.0.

### 4.2. Evaluation Indices

The primary reference record was the accuracy of the model forecast. In this paper, recall rate, specificity, and sensitivity were not used as evaluation indices. The classification performance was mainly evaluated by the classification accuracy, which was defined as follows:

(5) $$\begin{array}{c} \text{accuracy}=\frac{\text{TP}+\text{TN}}{\text{TP}+\text{TN}+\text{FP}+\text{FN}}, \end{array}$$

where TP, TN, FP, and FN denoted the true positive, the true negative, the false positive, and the false negative, respectively.

The loss function in the model was measured by the root mean square error, namely, a risk metric corresponding to the expected value of the squared (quadratic) error or loss. If ${\hat{y}}_{i}$ was the predicted value of the *i*-th sample and *y*_*i*_ was the corresponding true value, then the mean squared error (MSE) estimated over *n*_samples_ was defined as follows:

(6) $$\begin{array}{c} \text{MSE}\left(y,\hat{y}\right)=\frac{1}{n_{\text{samples}}}\sum_{i=0}^{n_{\text{samples}}-1}{\left(y_{i}-{\hat{y}}_{i}\right)}^{2}. \end{array}$$

### 4.3. Primary Learner Training Process and Results

**Figure 6:**
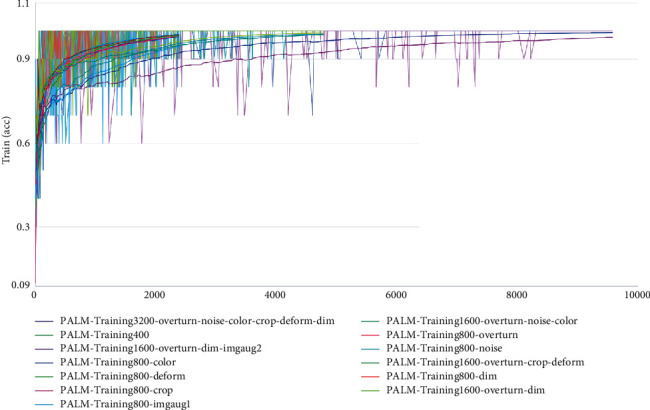
Accuracy of VGG-16 for 30 epochs of training on 13 datasets (original dataset: 2_num_ and DAMF datasets: 1_num_ and 3–13_num_).

As shown in Figure 6, firstly, VGG-16 was used as a dataset filter, and training was conducted on all datasets. Each dataset was trained for 30 epochs. Each epoch would traverse all the datasets once to form the corresponding trained models on different datasets. These 13 datasets were adopted to make predictions on the test set, and the final results are listed in Table 2. According to Table 2, the overall accuracy of the enhanced dataset was higher than that of the original dataset. To go into detail, the average accuracy of PALM-Training1600-overturning-dimming-imgaug2, PALM-Training3200-overturning-noise-color-cropping-deforming-dimming, PALM-Training1600-overturning-cropping-deforming, and PALM-Training800-color exceeded 95%. Therefore, these 4 datasets were selected to be the candidate datasets. GoogLeNet, AlexNet, and ResNet-50 were also trained on these 4 datasets. Each model was trained on each dataset for 30 epochs, and then the training model was tested on the test set.

Follow-up training was made on the candidate datasets, and the results are shown in Table 3. We used the same parameters during the process of training. It could be seen from Table 3 that the optimal DAMF datasets also varied because of the differences in the expression ability of different models. To be specific, GoogLeNet and ResNet-50 were both trained on the PALM-Training3200-overturning-noise-color-cropping-deforming-dimming dataset. AlexNet and VGG-16 had the highest scores on the PALM-Training1600-overturning-dimming-imgaug2 dataset. The above four models with the highest accuracy were used as primary learners.

In this paper, the accuracy rate was the average of the accuracy rate results of 30 epochs of training. The highest accuracy of the model training set reached 100% (Figure 6). Table 2 still shows the comparison between applying DAMF and not applying DAMF, in which the 13th dataset is the original dataset. We can see that the results of all the datasets processed by DAMF were better than the results of the 13th dataset, and the best DAMF corresponding to the first group of results is 2.84% higher than the 13th group, reached 95.85%. This improvement is obvious. Similarly, we have observed AlexNet, GoogLeNet, and ResNet-50. From the results for training, the accuracy rates of these were, respectively, 95.76%, 96.24%, and 95.60%, which are highly similar to the best training results of VGG-16, i.e., all exceed 95.00%. The above results could show that DAMF is universal.

**Figure 7:**
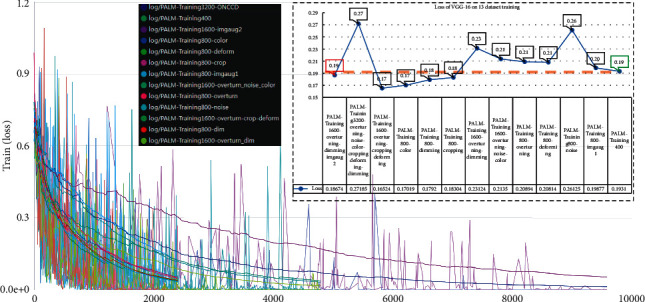
Loss of VGG-16 for 30 epochs of training on 13 datasets.

Figure 7 displays the loss trend during the training process of VGG-16. Taking the loss rate of the training original dataset as a baseline for comparison, we could find that although some DAMF loss rates were higher than 0.19, the highest loss rate was 0.27, which means the loss rate was acceptable. Among them, the loss rate corresponding to the best DAMF is 0.19, which was the same as the original dataset, which means that the enhanced data did not cause additional loss.

### 4.4. Fusion Model Training Process and Results

**Figure 8:**
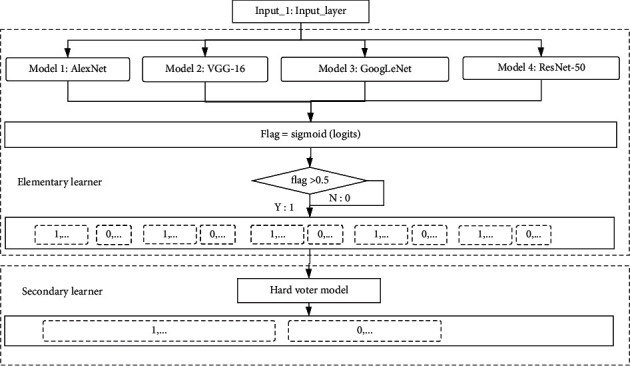
Logic diagram of the fusion model.

Figure 8 displays the logic diagram of the fusion model, with the most accurate models (AlexNet, GoogLeNet, ResNet-50, and VGG-16) used as the primary learners. The predictions of all primary learners were used as the training dataset for secondary learners. Meanwhile, the original dataset label was treated as the label of the new dataset to build the training set of the secondary model. After the primary model, the hard voting model was inserted as a classifier to form the framework of the secondary learners. In the secondary model, the four prediction results (AlexNet result, GoogLeNet result, ResNet-50 result, and VGG-16 result) in each sample were counted as the final prediction results. After 30 epochs of training, the model was saved and validated on a test set. The final accuracy of the fusion model reached 97.25% (average accuracy), with a maximum accuracy of 98.00%.

## 5. Discussion

First, 12 DAMF strategies were implemented on the iChallenge-PM dataset, resulting in the formation of 13 datasets, including the original one. Then, the experiment used VGG-16 as a dataset picker to train each of these 13 datasets for 30 epochs, each epoch covering all the data once. The accuracy of the model after training on each dataset was obtained on the validation set. As universally agreed, the four datasets with the best accuracy were selected as the preselected datasets to be used in the training of all remaining models. At the end of the training, the model with the highest prediction accuracy on the validation set was chosen as the primary learner.

Through this experiment, the advantages of DA were evident, and the data augmented datasets generally obtained higher accuracy than that of the original dataset. The average accuracy of the VGG-16 models trained by the four preselected datasets mentioned above was 95.85%, which was 2.84% higher than that of the original dataset.

This study adopted the strategy of model integration. The experiment retrained the output of the first-level model. The average accuracy of the first-level model was 95.86%, and the prediction accuracy of the fusion model was once again improved by 1.39%. Particularly, the greater significance of the fusion model was that the shortcomings of each primary learner were balanced. Therefore, the generalization performance and expression of the model were effectively improved.

**Figure 9:**
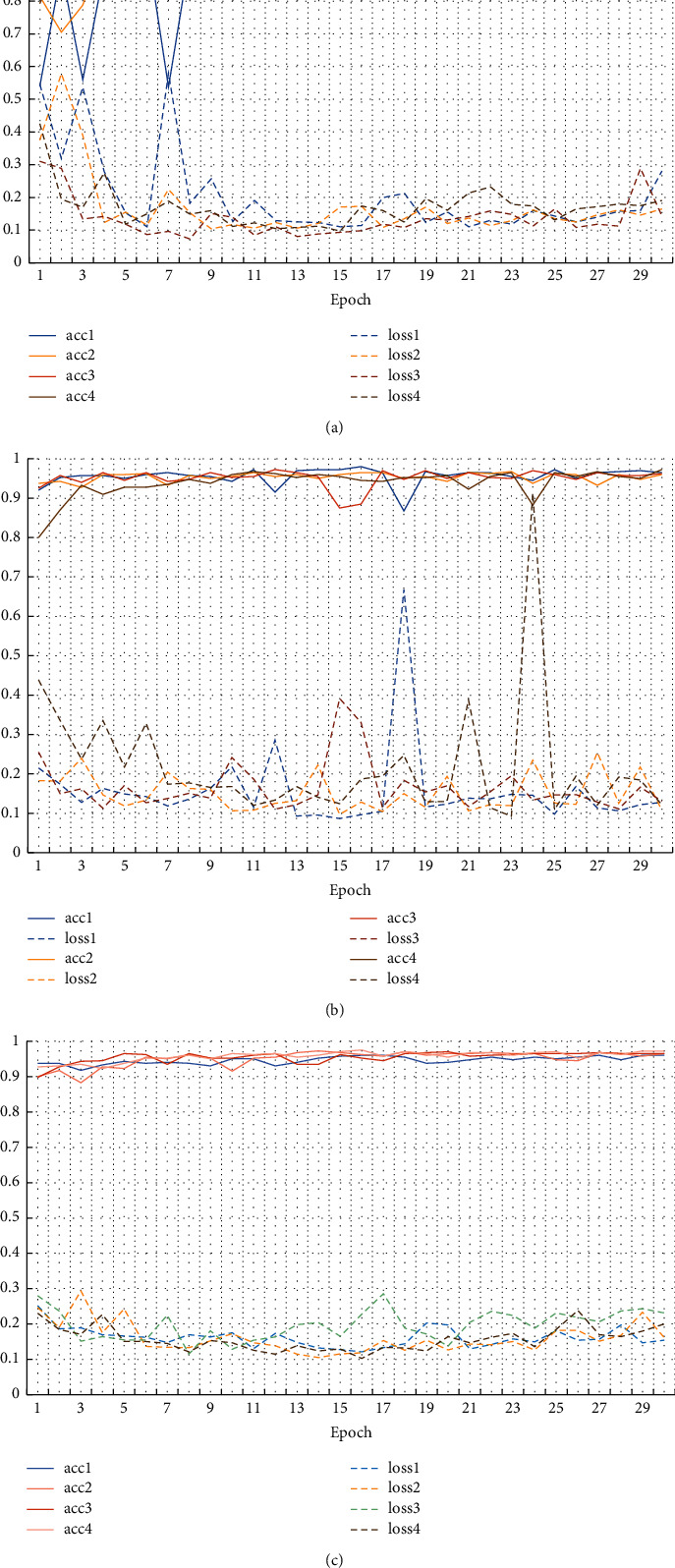
The accuracy and loss rate of three primary learners in training process. Four best training results of (a) AlexNet; (b) GoogLeNet; (c) ResNet-50.

We could observe the performance of DAMF in the primary learners again.  Figure 9 displays the training process of AlexNet, GoogLeNet, and ResNet-50 on the four optimal DAMF datasets. The details were expressed as follows: the solid line part in each subfigure referred to the accuracy, the dotted line part referred to the loss rate, the abscissa represented the training epoch, and the ordinate represented the percentage (%). It was worth noting that the accuracy of each model could reach about 95% at the end of training without model fusion, which meant that DAMF played a highly favorable role.

**Figure 10:**
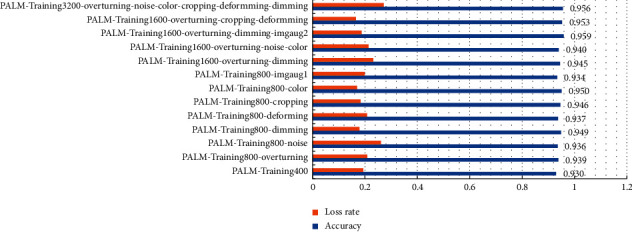
Distribution of accuracy and loss rate of VGG-16 under different MF.

We compared all DAMFs during the training process of VGG-16 by making them learn from all 13 datasets.  Figure 10 exhibits the training effect of VGG-16 on the passing accuracy and loss rate of each dataset. It was believed that DAMF should not be as complicated as possible. The best result appeared in PALM-Training1600-overturning-dimming-imgaug1. When DAMF got complicated, the effect would decrease instead. Excessively complex processing of images might destroy valuable features in the image. This meant that DA was a process rather than formula and that we needed to locate the DAMF dataset that best fit each dataset in the dynamic process.

We compared the classification results under different strategies on a fixed dataset (iChallenge-PM). Fully considering the differences of research studies, part of the research focused on the optimization of the network structure, while others focused on the DA. The overall idea of the research is to start from the optimization of the network model and DA direction, with the ultimate goal of model accuracy.  Table 4 displays the accuracy of different studies on the iChallenge-PM dataset in recent years. The results showed that although our accuracy rate is not the highest, it is also encouraging. In particular, our calculation cost was low. All training took 19 hours and 56 minutes, and no expensive calculation methods such as transfer learning were used.

## 6. Conclusion

The in-depth analysis of the discussed image DA techniques based on fusion has taken PM images as the research object and convincingly displayed their wide variety of applications. DAMF has the advantage of effectively improving the accuracy of model training, and the optimal enhanced set of different datasets can be matched through this strategy. By analyzing the image DA method proposed in this paper, DAMF proves to be better and more effective than other methods. The experiment results have shown that DA still has an optimal complexity in the combination of DAMF. Otherwise, too much complexity may destroy the original features. DAMF can effectively find the best combination of DA which well retains the characteristics of the input images and provides better contrast through 11 contrast combinations. It has also been observed that DAMF can train the model to satisfactory results without using transfer learning or other methods. Arguably, DAMF can be used as an effective DA method during the training of CNNs in the field of fundus image processing.

## Figures and Tables

**Algorithm 1:**
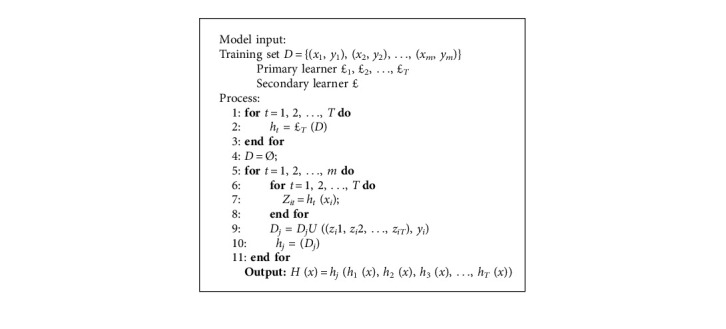
Logic diagram of the model fusion algorithm.

**Table 1:** 12 DAMFs.

No.	Training set name	DA method	Quantity
1	PALM-Training800-overturning	Original dataset + random flip (4 directions: up, down, left, and right)	800
2	PALM-Training800-noise	Original dataset + Gaussian white noise	800
3	PALM-Training800-color	Original dataset + randomly changing colors (brightness, contrast, saturation)	800
4	PALM-Training800-cropping	Original dataset + random cropping	800
5	PALM-Training800-deforming	Original dataset + random scaling, stretching (stretched into a square by the length or width of the images)	800
6	PALM-Training800-dimming	Original dataset + change clarity	800
7	PALM-Training1600-overturning-noise-color	Randomly stack method 3 or 4 (serial number) on the basis of PALM-Training800-overturning	1600
8	PALM-Training1600-overturning-cropping-deforming	Randomly stack method 5 or 6 (serial number) on the basis of PALM-Training800-overturning	1600
9	PALM-Training1600-overturning-dimming	Randomly stack method 7 (serial number) on the basis of PALM-Training800-overturning	1600
10	PALM-Training3200-overturning-noise-color-cropping-deforming-dimming	Randomly superimpose method 5 or 6 or 7 (serial number) on the basis of PALM-Training800-overturning-noise-color	3200
11	PALM-Training800-imgaug1	Original dataset + random cropping with 0–50 pixels around, 50% probability horizontal flip, Gaussian blur (sigma = 0 to 3.0)	800
12	PALM-Training1600-overturning-dimming-imgaug2	PALM-Training800-overturning-dimming dataset + multiple mixed random overlay	1600

**Table 2:** VGG-16 training results on 13 datasets.

No.	Dataset	Accuracy	Loss
1	PALM-Training1600-overturning-dimming-imgaug2	0.95858336	0.18674079
2	PALM-Training3200-overturning-noise-color-cropping-deforming-dimming	0.95550001	0.27185006
3	PALM-Training1600-overturning-cropping-deforming	0.95266668	0.16523545
4	PALM-Training800-color	0.95033336	0.17019135
5	PALM-Training800-dimming	0.94875002	0.17919912
6	PALM-Training800-cropping	0.94625	0.18303553
7	PALM-Training1600-overturning-dimming	0.94525003	0.23124305
8	PALM-Training1600-overturning-noise-color	0.94008333	0.21350351
9	PALM-Training800-overturning	0.93858335	0.20894363
10	PALM-Training800-deforming	0.93708334	0.20814224
11	PALM-Training800-noise	0.93608335	0.26124661
12	PALM-Training800-imgaug1	0.93391667	0.19876853
13	**PALM-Training400**	**0.93016667**	0.19310093

**Table 3:** Training results of VGG-16, AlexNet, GoogLeNet, and ResNet-50 on the filtered datasets.

Primary learner	PALM-Training800-color		PALM-Training1600-overturning-cropping-deforming		PALM-Training3200-overturning-noise-color-cropping-deforming-dimming		PALM-Training1600-overturning-dimming-imgaug2	
	Accuracy	Loss rate	Accuracy	Loss rate	Accuracy	Loss rate	Accuracy	Loss rate
AlexNet	0.946083	0.162323	0.950833	0.160698	0.954667	0.197096	0.957583	0.157773
GoogLeNet	0.909	0.203461	0.9395	0.169759	0.962417	0.13617727	0.947917	0.170418
ResNet-50	0.9395	0.219571	0.953583	0.151836	0.955917	0.156737	0.95125	0.166358
VGG-16	0.9503	0.17019	0.95267	0.16523	0.9555	0.27185	0.95858	0.18674

**Table 4:** Results on the iChallenge-PM dataset.

	Accuracy (%)	Methods
Siying Dai [28]	81.82	Optimize network structure + DA
InstDis [48]	95.32	Optimize network structure + DA
Contrastive [49]	96.94	Optimize network structure + DA
Invariant [50]	97.30	Optimize network structure + DA
Xiaomeng Li [51]	98.65	Optimize network structure + DA
**Ours**	**97.25**	Optimize network structure + DA

## Data Availability

The data used to support the findings of this study are included within the article.
